# Association of *MAX* Copy Number Variation with Morphometric Traits in Chinese Cattle

**DOI:** 10.3390/ani16152406

**Published:** 2026-08-04

**Authors:** Shiyi Lv, Boyu Li, Suyun Fan, Xiangnan Wang, Nan Liu, Xiukai Cao, Ping Qian, Jie Cheng

**Affiliations:** 1 Jiangsu Key Laboratory of Sericultural and Animal Biotechnology, School of Biotechnology, Jiangsu University of Science and Technology, Zhenjiang 212100, China; lvshiyi2005@163.com (S.L.); 15831277690@163.com (B.L.); fanwyyx163@163.com (S.F.); qianping@just.edu.cn (P.Q.); 2 Key Laboratory of Silkworm and Mulberry Genetic Improvement, Ministry of Agriculture and Rural Affairs, Sericultural Scientific Research Center, Chinese Academy of Agricultural Sciences, Zhenjiang 212100, China; 3 Institute of Animal Husbandry and Veterinary Science, Henan Academy of Agricultural Sciences, Zhengzhou 450002, China; 18838917969@163.com; 4 Animal Disease Prevention and Control Center of Xizang Autonomous Region (Animal Husbandry Station of Xizang Autonomous Region), Lhasa 850000, China; 5 Joint International Research Laboratory of Agriculture and Agri-Product Safety of Ministry of Education of China, Yangzhou University, Yangzhou 225009, China

**Keywords:** copy number variation, *MAX*, Chinese cattle, association analysis, morphometric traits

## Abstract

Copy number variation (CNV) is a major class of genomic structural variation that can shape economically important traits by altering gene dosage and gene expression. *MAX* (*MYC-associated factor X*), a core component of the *MYC*-*MAX*-*MXD1* transcriptional regulatory network, is critically involved in cell proliferation, differentiation, and development. However, the relationship between *MAX* CNVs and morphometric traits in cattle remains largely unknown. Here, we genotyped *MAX* CNVs by quantitative real-time PCR (qPCR) in 572 Chinese cattle from five breeds, including Qinchuan (QC), Ji’an (JA), Jinnan (JN), Nanyang (NY), and Xianan (XN) cattle, and evaluated the association of *MAX* CNVs with morphometric traits. *MAX* CNVs exhibited distinct breed-specific distribution patterns: the gain type predominated in QC, the loss type predominated in JN, NY, and XN, whereas JA showed a relatively balanced distribution across the three CNV types. Based on raw *p* values, *MAX* CNVs showed a nominal association with chest girth in JN cattle and nominal associations with chest girth and hucklebone width in NY cattle (*p* < 0.05). Individuals with the medium type showed higher least-squares means for the nominally associated traits than those with the loss or gain types. *MAX* CNVs explained 5.9% of the phenotypic variance in chest girth in JN cattle and 13.6% and 12.2% of the phenotypic variance in chest girth and hucklebone width in NY cattle, respectively. These results suggest that *MAX* CNVs may represent a breed-specific candidate locus related to morphometric traits in Chinese cattle. However, because no formal multiple-testing correction was applied and this study did not include independent population validation or functional validation, further studies are needed before considering any potential application in marker-assisted selection.

## Simple Summary

Morphometric traits are important in beef cattle breeding because they directly affect meat production and economic value. Differences in these traits among cattle breeds are partly influenced by genetic variation. In this study, we examined copy number variation (CNV) in the *MYC-associated factor X* gene in 572 Chinese cattle from five breeds, including Qinchuan, Ji’an, Jinnan, Nanyang, and Xianan cattle. CNVs are defined as differences in the number of copies of genomic DNA segments among individuals within a population. The copy number variation in this gene showed different distribution patterns among the five breeds. Based on raw *p* values, *MAX* CNVs showed nominal associations with chest girth in Jinnan cattle and with chest girth and hucklebone width in Nanyang cattle. Animals with the medium copy type, defined as two copies, showed higher values for the nominally associated traits than those with the loss type (<2 copies) or gain type (>2 copies). These findings suggest that copy number variation in the *MYC-associated factor X* gene may represent a potential candidate locus related to morphometric traits in Chinese cattle. Further validation in larger cattle populations is still needed before it can be used in breeding programs.

## 1. Introduction

Morphometric traits directly determine production efficiency and profitability in the beef cattle industry and are therefore of great economic importance in livestock production. Chinese cattle breeds show substantial differences in genetic background, body conformation, growth rate, and meat production performance. In the present study, five representative Chinese cattle breeds were selected for analysis, including Qinchuan cattle (QC), Ji’an cattle (JA), Jinnan cattle (JN), Nanyang cattle (NY), and Xianan cattle (XN). QC is a typical breed of the Central Plains cattle group, characterized by large body size and fine meat quality but relatively slow growth. NY has both beef and draft advantages, with a robust skeletal structure and well-developed musculature. JN is adapted to roughage feeding and harsh working conditions and has favorable potential for both draft and meat production. JA possesses unique genetic potential in meat quality traits and local adaptation. XN is a crossbred breed developed from Nanyang cattle and Charolais cattle and exhibits superior growth rate and meat production efficiency compared with indigenous Chinese cattle [1]. Collectively, these breeds represent valuable genetic resources for the Chinese cattle industry. Nevertheless, relatively slow growth and limited meat yield remain major constraints in many Chinese cattle populations, highlighting the need to identify genetic markers for molecular-assisted breeding and genetic improvement in Chinese cattle.

Copy number variation (CNV) is an important class of genomic structural variation, generally defined as differences in the number of copies of genomic DNA segments among individuals within a population, typically involving segments ranging from approximately 50 bp to 5 Mb. CNVs mainly include deletions and duplications, leading to copy-number losses or gains of genomic segments. Compared with single nucleotide polymorphisms (SNPs) and small insertions/deletions (indels), CNVs usually span larger genomic regions, occur at higher mutation rates, and may involve variable breakpoints and copy-number states [2]. Functionally, CNVs can directly alter gene dosage [3] and can also modulate gene expression through disruption or repositioning of regulatory elements [4], gene fusion [5], and changes in three-dimensional chromatin organization [6]. Therefore, CNVs can have profound effects on animal growth, reproduction, disease resistance, and domestication-related adaptation. With the rapid development of molecular breeding, CNVs have become important molecular markers for marker-assisted selection and genomic breeding. CNV detection has advanced from early array-based comparative genomic hybridization (aCGH) [7] and SNP arrays [2] to high-resolution approaches based on next-generation sequencing (NGS) and third-generation sequencing (TGS) [8], together with targeted validation methods such as qPCR, fluorescence in situ hybridization (FISH), and multiplex ligation-dependent probe amplification (MLPA) [9,10,11]. Current CNV studies mainly focus on association analysis and functional validation. Candidate CNV regions associated with growth, meat quality, reproduction, milk production, disease resistance, and high-altitude adaptation are usually identified by integrating genome-wide association studies (GWASs), quantitative trait locus (QTL) mapping, and population genetic analyses, followed by qPCR, expression analysis, and gene-editing approaches to evaluate their functional relevance [12]. In cattle, CNVs show marked breed-specific distributions and have been associated with multiple economically important traits, such as *WC1* with parasite resistance [13], *USP16* with milk production traits [14], and *ZNF280BY* with bull fertility [15]. These findings underscore the potential value of CNVs in livestock domestication studies, breed identification, and molecular breeding. However, whether CNVs exist in the *MYC-associated factor X* (*MAX*) gene and whether it is associated with economically important traits in cattle remain unknown.

The *MAX* gene encodes a nuclear transcriptional regulatory protein and is a core component of the *MYC-MAX-MXD* regulatory network, also known as the *Myc/Max/Mad* network. In this network, *MAX* forms heterodimers with *MYC* or *MXD* family members, thereby participating in transcriptional activation or repression. Previous studies have shown that the *Myc/Max/Mad* network regulates key cellular processes, including cell proliferation, differentiation, apoptosis, cell-cycle progression, growth-related cellular behavior, and morphology [16,17,18]. These biological processes are closely related to animal growth, body conformation, and tissue development [19]. In the bovine genome, a CNV is located in the upstream region of *MAX* at chr10:77227001–77230500 (bosTau9), spanning approximately 3.5 kb [20]. Because upstream regions may contain regulatory elements, this CNV represents a positional candidate variant. However, no direct evidence is currently available to show that this upstream CNV overlaps experimentally validated regulatory elements, has regulatory activity, or affects *MAX* expression; therefore, its biological importance remains to be validated through regulatory annotation, expression analysis, and functional experiments. Previous studies have reported that *MXD3*, a gene involved in the same regulatory network, is associated with body weight and withers height in cattle [21]. Therefore, although the function of *MAX* CNVs in cattle has not yet been experimentally validated, the biological role of the *MYC-MAX-MXD* network provides a rationale for investigating whether the CNV near *MAX* is associated with morphometric traits. To our knowledge, *MAX* CNVs have not previously been characterized in these Chinese cattle populations. Therefore, this study aimed to describe the breed-specific distribution of *MAX* CNVs in QC, JA, JN, NY, and XN cattle and to explore its association with morphometric traits.

## 2. Materials and Methods

### 2.1. Study Populations and Trait Records

A total of 572 cattle from five Chinese breeds were genotyped in this study, including Qinchuan cattle (QC, n = 79), Ji’an cattle (JA, n = 77), Jinnan cattle (JN, n = 120), Nanyang cattle (NY, n = 90), and Xianan cattle (XN, n = 206). These animals were raised in Shaanxi, Jiangxi, Shanxi, Henan, and Henan provinces, respectively. The cattle were weaned at 6 months of age and were provided ad libitum access to concentrate, corn silage, and straw until approximately 2 years of age, at which point they were considered adults in this study. All selected individuals were unrelated for at least three generations. The sample size was determined by the availability of cattle populations with genotyping data and corresponding phenotypic records; no a priori sample size calculation was performed. This was an observational genetic association study, and the association analyses were based on available phenotypic records within each breed.

Morphometric traits were measured according to standardized procedures described previously [22]. Body weight was recorded using a livestock scale, whereas linear body measurements were obtained using a measuring stick or measuring tape, depending on the trait. Measurements were performed by trained personnel following the same measurement criteria within each population to reduce measurement error. Among the 572 genotyped cattle, the availability of phenotypic records differed among breeds because the original trait records were not equally complete across populations. Usable phenotypic data were available for 76 QC cattle, 56 JA cattle, 113 JN cattle, and 178 XN cattle. For NY cattle, repeated trait records were collected at six age stages from birth to 3 years of age; therefore, a *MAX*imum of 540 records could be obtained from 90 animals, and 203 usable age-stage records were included in the association analysis after data filtering. Because phenotypic data collection differed among breeds, the association analyses were interpreted within each breed rather than as direct comparisons of phenotypic values across breeds.

In QC, withers height, thurl width, hip width, body length, chest girth, chest width, chest depth, rump length, hucklebone width, and body weight were recorded in adult female cattle. In JA, withers height, hip width, body oblique length, chest girth, rump length, and cannon bone circumference were recorded in female cattle aged approximately 8 months to 2.5 years. In JN, withers height, hip width, body oblique length, chest girth, rump length, and body weight were recorded in adult cattle with available phenotypic data. In NY, body weight, withers height, body oblique length, chest girth, and hucklebone width were recorded at birth, 6 months, 1 year, 1.5 years, 2 years, and 3 years. In XN, withers height, hip width, body oblique length, chest girth, waist-abdominal circumference, cannon bone circumference, and body weight were recorded in adult females and castrated males. Among these traits, hucklebone width refers to the distance between the left and right ischial tuberosities. A descriptive summary of the raw phenotypic data, including the number of records, minimum, maximum, mean, median, and SD values for each breed, age stage, and morphometric trait, is provided in Supplementary Table S1.

### 2.2. Genomic DNA Isolation

Genomic DNA was extracted from 1 mL of whole blood treated with 2% heparin using the standard phenol–chloroform method. Briefly, leukocytes were isolated and lysed with proteinase K, and the samples were then treated with Tris-saturated phenol and chloroform to remove proteins. DNA was subsequently precipitated with absolute ethanol, collected by centrifugation, and washed with 70% ethanol. The genomic DNA samples were assessed using a spectrophotometer, diluted to 50 ng/μL, and stored at −80 °C until further analysis.

### 2.3. Determination of MAX Gene Copy Numbers

In this study, qPCR was used to genotype *MAX* CNVs because of its high sensitivity and specificity for relative quantification of target loci [23]. In the qPCR assay, the bovine *basic transcription factor 3* (*BTF3*) gene was used as a diploid internal reference gene because it has been reported to have a stable copy number in the bovine genome, enabling reliable normalization of relative *MAX* CNV measurements [24]. Primers were designed within the CNV region based on sequences retrieved from the NCBI database (Table 1).

qPCR was performed using 2× ChamQ Blue Universal SYBR qPCR Master Mix on a QuantStudio™ 3 Real-Time PCR Instrument (96-well, 0.2 mL block; Life Technologies Holdings Pte Ltd., Singapore). Each 10 μL reaction contained 5 μL of 2× ChamQ Blue Universal SYBR qPCR Master Mix, 0.5 μL each of forward and reverse primers, 3 μL of ddH_2_O, and 1 μL of genomic DNA. The thermal cycling conditions were as follows: initial denaturation at 95 °C for 30 s, followed by 40 cycles of 95 °C for 10 s and 60 °C for 30 s. Primer specificity was verified by melting curve analysis and no-template control (NTC) reactions. Copy number was calculated using the 2^−ΔCt^ method, and the values were rounded to the nearest integer. Because cattle are diploid, the estimated relative copy numbers were rounded to the nearest integer and then classified into three CNV types: loss type (copy number < 2), medium type (copy number = 2), and gain type (copy number > 2).

### 2.4. Statistical Analyses

Association analyses between the *MAX* CNV type and morphometric traits were performed separately within each breed rather than by pooling all breeds, because the five breeds differed in genetic background, CNV distribution, age structure, sex composition, available phenotypic records, and recorded traits. Therefore, breed was not included as a fixed effect in the final model, because breed did not vary within each breed-specific analysis and could not be estimated as an additional effect. Farm was also not included in the final breed-specific models when it did not vary within breed. No pooled cross-breed association analysis was performed, which reduced potential confounding from among-breed population structure.

For each trait, a general linear model was fitted with the *MAX* CNV type as the main fixed effect. Age, sex, birth season, farm, and sire were initially considered as potential covariates or fixed effects when the corresponding information was available. Age was treated as an age or age-stage effect when retained in the model, rather than assuming a common linear age effect across all breeds and traits. Because all JN cattle included in the analysis were adults, the age term was not retained in the final JN breed-trait models. The final model for each breed-trait analysis was determined according to data availability, biological relevance, within-breed variation, and model estimability. Covariates were retained when the corresponding information was sufficiently available and could be reliably estimated; otherwise, they were omitted from the final model. Therefore, the final model varied among breeds and traits according to the availability, biological relevance, and estimability of covariates. The general form of the model was as follows:

*Y_ijkl_* = *μ* + *A_i_* + *S_j_* + CNV*_k_* + *e_ijkl_* (1)

where *Y_ijkl_* represents the observed value of a morphometric trait, μ is the overall mean of each trait, *A_i_* is the effect of age or age stage, *S_j_* is the effect of sex, CNV*_k_* is the fixed effect of the kth *MAX* CNV type, and *e_ijkl_* is the random residual error. Because phenotypic records, age structure, and sex composition differed among breeds, the age and sex terms were included only when the corresponding information was sufficiently available, varied within the breed, and could be reliably estimated; otherwise, these terms were omitted from the final breed-trait model. For each fitted model, the residual error term was assumed to be normally distributed with a mean of zero and a constant variance, i.e., *e_ijkl_* ~ N(0, σe^2^). Sire effect was not included in the final model because all animals were unrelated for at least three generations.

Association analyses between the *MAX* CNV type and morphometric traits were performed using a general linear model in SPSS 27.0. The proportion of phenotypic variance explained by the *MAX* CNV type was estimated using partial correlation analysis in SPSS 27.0. No animals or data points were excluded from the analysis after quality control unless phenotypic or genotyping data were unavailable. Raw *p* values are reported in the association analyses. Because no formal multiple-testing correction was applied, associations with raw *p* < 0.05 were considered nominal associations and were interpreted cautiously.

## 3. Results

### 3.1. CNV Polymorphisms of MAX in Five Chinese Cattle Breeds

**Figure 1:**
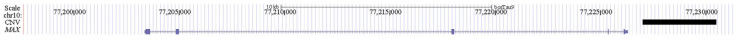
Schematic diagram of the *MAX* CNV region (chr10:77227001–77230500, bosTau9, ~3.5 kb) located upstream of the bovine *MAX* gene. The diagram was generated using the UCSC Genome Browser based on the bosTau9 assembly and the CNV coordinates reported by Huang et al. [20].

**Figure 2:**
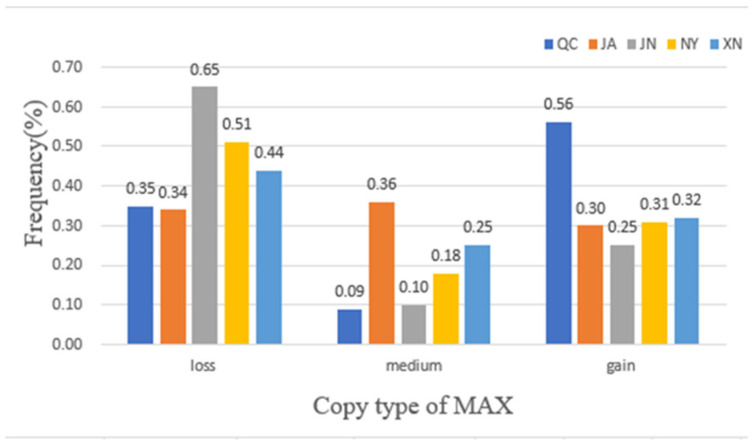
Distribution of *MAX* CNV types in five Chinese cattle breeds: QC (n = 79), JA (n = 77), JN (n = 120), NY (n = 90), XN (n = 206).

qPCR-based genotyping confirmed the presence of *MAX* CNV polymorphisms at chr10:77227001–77230500 (bosTau9) in all five Chinese cattle breeds examined (Figure 1). *MAX* CNV polymorphisms were detected in all five breeds (Figure 2). The distribution of *MAX* CNVs differed markedly among the five breeds (Figure 2). Specifically, the gain type was predominant in QC (56%), whereas the loss type was predominant in JN (65%), NY (51%), and XN (44%). In contrast, JA showed a relatively balanced distribution, with loss, medium, and gain types accounting for 34%, 36%, and 30%, respectively. These results indicate that the upstream CNV region of *MAX* exhibits a clear breed-specific distribution pattern in Chinese cattle.

### 3.2. Association Between MAX CNV and Morphometric Traits

Association analyses revealed no associations between the *MAX* CNV type and morphometric traits in QC, JA, or XN cattle based on raw *p* values (*p* > 0.05). In JN cattle, the *MAX* CNV type showed a nominal association with chest girth based on the raw *p* value (*p* < 0.05). In NY cattle, the *MAX* CNV type showed nominal associations with chest girth and hucklebone width based on raw *p* values (*p* < 0.05). Individuals with the medium type showed higher values for the nominally associated traits than those with the loss or gain types (Table 2).

### 3.3. Phenotypic Variance Explained by MAX CNV

For traits showing nominal associations based on raw *p* values, *MAX* CNVs explained 13.6% and 12.2% of the phenotypic variance in chest girth and hucklebone width in NY cattle, respectively, and 5.9% of the phenotypic variance in chest girth in JN cattle (Table 3).

## 4. Discussion

This study characterized the distribution of *MAX* CNVs in five Chinese cattle breeds and explored its association with morphometric traits. Based on raw *p* values, nominal associations were detected for chest girth in JN cattle and for chest girth and hucklebone width in NY cattle. However, these findings should be interpreted cautiously, as reliance on significance-based model reduction may introduce bias and inflate Type I error rates. Because the identified CNV is located upstream of *MAX*, it may represent a positional candidate variant related to regulatory variation; however, its regulatory activity and effect on *MAX* expression remain to be experimentally validated.

In this study, *MAX* CNV polymorphisms were detected in all five Chinese cattle breeds examined. *MAX* CNVs showed distinct breed-specific distribution patterns, with the gain type predominating in QC and the loss type in JN, NY, and XN, while JA showed a balanced distribution. This distribution pattern suggests that the upstream CNV region of *MAX* may be shaped by differences in genetic background, artificial selection, natural selection, and population history among cattle breeds. This finding is consistent with previous observations for *CCDC39* CNV, in which the predominant copy-number type varied among cattle breeds, supporting the view that the CNV distribution can be population- or breed-specific [26].

Association analysis further revealed that *MAX* CNVs showed nominal associations with chest girth in JN cattle and with chest girth and hucklebone width in NY cattle. Individuals with the medium type showed better phenotypic performance for the nominally associated traits than those with the loss or gain types, suggesting that copy-number changes in this region may affect *MAX* transcriptional regulation and downstream biological processes related to growth. Because the identified CNV is located upstream of *MAX*, it may represent a positional candidate variant related to regulatory variation. However, whether this CNV affects transcription factor binding, chromatin accessibility, enhancer activity, *MAX* expression, or downstream pathways remains unknown and requires further validation through expression analysis and functional experiments.

For traits showing nominal associations based on raw *p* values, *MAX* CNVs accounted for 13.6% and 12.2% of the phenotypic variance in chest girth and hucklebone width in NY cattle, respectively, and 5.9% of the phenotypic variance in chest girth in JN cattle under the current statistical model. Chest girth is an important indicator of body size and thoracic development, whereas hucklebone width reflects posterior skeletal conformation. Thus, the nominal associations observed in JN and NY cattle suggest that *MAX* CNVs may represent a breed-specific candidate locus related to morphometric traits. Nevertheless, no nominal association was detected in QC, JA, or XN cattle, indicating that the phenotypic effect of *MAX* CNVs may be dependent on breed-specific genetic background, selection history, environmental conditions, and population structure. In addition, the relatively limited sample size in some groups may have reduced the statistical power to detect weak or moderate genetic effects. Moreover, no formal multiple-testing correction was applied; therefore, associations based on raw *p* values should be interpreted as nominal associations and require validation in larger independent populations. Because qPCR-based copy-number estimation may be affected by technical variation, samples with estimated values close to classification boundaries may have classification uncertainty. Therefore, further validation using ddPCR or sequencing-based methods would be useful in future studies. Although breed-specific analyses were used to reduce confounding from among-breed population structure, residual population stratification within breeds and unmeasured family structure cannot be completely excluded. In addition, differences in age structure, sex composition, and recorded morphometric traits among breeds may affect the comparability of results across breeds and should be considered when interpreting the breed-specific findings.

Overall, this study provides preliminary evidence that *MAX* CNVs may represent a potential positional candidate locus associated with morphometric traits in Chinese cattle based on raw *p* values, particularly chest girth in JN cattle and chest girth and hucklebone width in NY cattle. However, because this study was based primarily on candidate-gene association analysis, the causal relationship between *MAX* CNVs and morphometric traits remains to be established. Future studies should include larger and independent populations, *MAX* expression profiling, regulatory annotation of the CNV region, and functional validation to clarify the molecular mechanism by which this CNV may influence cattle morphometric traits.

## 5. Conclusions

This study characterized the distribution of *MAX* CNVs across five Chinese cattle breeds and evaluated their association with morphometric traits. *MAX* CNVs displayed breed-specific distribution patterns. Based on raw *p* values, *MAX* CNVs showed a nominal association with chest girth in JN cattle and nominal associations with chest girth and hucklebone width in NY cattle. Individuals with the medium type showed higher least-squares means for the nominally associated traits than those with the loss or gain types. These findings suggest that *MAX* CNVs may represent a potential breed-specific positional candidate locus related to morphometric traits in Chinese cattle. However, because no formal multiple-testing correction was applied, these nominal associations should be interpreted cautiously. Further validation in larger independent populations, together with expression and functional analyses, is required before considering its application in marker-assisted selection.

## Figures and Tables

**Table 1:** PCR primer sequences of the cattle *MAX* gene for qPCR in this study.

Name of CNV	Primers Pair	Primers (5′-3′)	Product Size (bp)
*MAX*-CNV	P1	F:GTCTCAGTCTTCCTTTCACACCA R:ACTCGTCCAAGAACTACCTACCT	102
*BTF3*-CNV	P2	F:CAAGAAGACTCATTCCTT R:CACAAGCACATTATTCAC	109

**Table 2:** Association between *MAX* CNV types and morphometric traits in QC, JA, JN, NY, and XN cattle.

Breeds	Morphometric Traits	CNV Types (LSM ± SE)			*p* Value
		Loss	Medium	Gain	
QC	Withers height (cm)	130.531 ± 1.560	128.520 ± 2.245	129.983 ± 1.308	0.725
	Hip width	127.753 ± 1.534	125.748 ± 2.208	127.715 ± 1.286	0.694
	Body length	138.317 ± 2.486	142.752 ± 3.577	140.056 ± 2.083	0.508
	Chest girth	186.890 ± 6.540	187.169 ± 9.409	179.335 ± 5.481	0.401
	Chest width	40.634 ± 1.517	40.496 ± 2.182	40.275 ± 1.271	0.967
	Chest depth	65.923 ± 1.492	64.536 ± 2.147	63.397 ± 1.250	0.190
	Rump length	44.665 ± 1.294	43.874 ± 1.862	43.079 ± 1.085	0.413
	Hucklebone width	23.865 ± 1.337	25.798 ± 1.924	23.669 ± 1.121	0.588
	Thurl width	39.407 ± 12.703	41.805 ± 18.277	55.207 ± 10.646	0.374
	Body weight	447.524 ± 20.407	465.445 ± 29.362	433.187 ± 17.104	0.509
JA	Cannon bone circumference	13.289 ± 0.270	13.525 ± 0.257	13.332 ± 0.255	0.521
	Withers height (cm)	101.524 ± 2.301	99.858 ± 2.192	99.653 ± 2.174	0.552
	Hip width	98.293 ± 6.293	102.985 ± 5.996	101.815 ± 5.947	0.630
	Body oblique length	111.506 ± 3.157	109.746 ± 3.008	109.144 ± 2.984	0.640
	Chest girth	136.331 ± 4.230	141.028 ± 4.030	137.702 ± 3.997	0.375
	Rump length	163.615 ± 5.358	168.527 ± 5.105	165.267 ± 5.063	0.516
JN	Body weight	465.702 ± 26.704	519.539 ± 36.435	440.846 ± 31.949	0.083
	Withers height (cm)	127.491 ± 1.732	129.332 ± 2.363	125.865 ± 2.072	0.279
	Hip width	120.013 ± 2.006	130.338 ± 2.737	128.839 ± 2.400	0.837
	Body oblique length	149.359 ± 3.111	151.538 ± 4.245	145.979 ± 3.722	0.296
	Chest girth	182.622 ^b^ ± 3.856	192.092 ^a^ ± 5.262	179.444 ^b^ ± 4.614	**0.046**
	Rump length	47.586 ± 1.300	48.437 ± 1.809	46.023 ± 1.570	0.271
NY	Body weight	255.064 ± 2.947	258.940 ± 5.637	248.388 ± 4.227	0.245
	Withers height (cm)	119.955 ± 0.640	121.006 ± 1.231	119.671 ± 0.925	0.657
	Body oblique length	126.855 ± 0.698	129.907 ± 1.343	125.279 ± 1.009	0.083
	Chest girth	155.876 ^a^ ± 0.907	156.378 ^ab^ ± 1.746	152.259 ^b^ ± 1.312	**0.043**
	Hucklebone width	23.441 ^A^ ± 0.211	24.273 ^A^ ± 0.407	22.682 ^B^ ± 0.306	**0.005**
XN	Withers height (cm)	137.227 ± 0.617	136.564 ± 0.789	136.963 ± 0.714	0.761
	Hip width	140.019 ± 0.510	140.388 ± 0.652	139.656 ± 0.590	0.647
	Body oblique length	159.415 ± 0.961	159.366 ± 1.228	158.997 ± 1.112	0.943
	Chest girth	206.931 ± 3.715	202.264 ± 4.747	203.063 ± 4.299	0.611
	Paunch girth	216.067 ± 2.464	219.061 ± 3.322	214.646 ± 2.754	0.590

LSM, least-squares mean; SE, standard error. Associations with raw *p* < 0.05 are shown in bold and should be interpreted as nominal associations because no formal multiple-testing correction was applied. Different letters in the same row indicate significant differences among CNV types; different lowercase letters indicate *p* < 0.05, whereas different uppercase letters indicate *p* < 0.01 [25]. The numbers of available records in each CNV type were as follows: QC, loss = 27, medium = 7, gain = 42; JA, loss = 20, medium = 19, gain = 17; JN, loss = 71, medium = 12, gain = 30; NY, loss = 115, medium = 31, gain = 57; XN, loss = 76, medium = 44, gain = 58.

**Table 3:** Proportion of the phenotypic variance explained by *MAX* CNV in NY and JN cattle.

Breed	Trait	Proportion of Variance Explained (R^2^)
NY	Chest girth	13.6%
	Hucklebone width	12.2%
JN	Chest girth	5.9%

## Data Availability

The data supporting the findings of this study are available from the corresponding author upon reasonable request.

## Supplementary Materials

The following supporting information can be downloaded at: https://www.mdpi.com/article/10.3390/ani16152406/s1, Table S1: Descriptive statistics of raw phenotypic data for morphometric traits in different cattle breeds.

## Abbreviations

The following abbreviations are used in this manuscript:

CNV	Copy number variation
*MAX*	*MYC**-associated factor X*
qPCR	quantitative real-time PCR
QC	Qinchuan
JA	Ji’an
JN	Jinnan
NY	Nanyang
XN	Xianan
